# Supplementary material for: Histo-Blood Group Antigen-Producing Bacterial Cocktail Reduces Rotavirus A, B, and C Infection and Disease in Gnotobiotic Piglets
Source: Viruses. 2024 Apr 24;16(5):660. doi: 10.3390/v16050660 (PMC11125826; doi:10.3390/v16050660)
Supplement: Supplementary file 1 [file viruses-16-00660-s001.zip › viruses-2969712-supplementary.pdf]

viruses

Article

Histo-blood group antigen-producing bacteria cocktail reduces rotavirus A, B and C infection and disease in gnotobiotic piglets

Sergei A. Raev[1], Maryssa K. Kick[1], Maria Chellis[1], Joshua O. Amimo[2], Linda J. Saif[1], and Anastasia N. Vlasova[1]*

[1] Center for Food Animal Health, Department of Animal Sciences, College of Food, Agricultural and Environmental Sciences, The Ohio State University, Wooster, OH, USA
[2] GIVAX Inc., Philadelphia, PA, USA
* Correspondence: vlasova.1@osu.edu;

SUPPLEMENTARY MATERIALS

Table S1. Primers used in this study.

Target Organism	Primer	Sequence (5′-3′)	Reference
S. bovis	**F**	ATGTTAGATGCTTGAAAGGAGCAA	(43)
	**R**	CGCCTTGGTGAGCCGTTA	
B. thetaiotaomicron	**F**	CGTTCCATTAGGCAGTTGGT	(44)
	**R**	CGTAGGAGTTTGGACCGTGT	
C. clostridiiforme	**F**	AATCTTGATTGACTGAGTGGCGGAC	(45)
	**R**	CCATCTCACACTACCGGAGTTTTTC	
B. adolescentis	**F**	CTCCGCCGCTGATCCGGAAGTCG	(46)
	**R**	AACCAACTCGGCGATGTGGACGACA	
B. longum	**F**	CGGCGTYGTGACCGTTGAAGAC	
	**R**	TGYTTCGCCRTCGACGTCCTCA	
L. brevis	**F**	CTTCTGGATGATCCCGCGGCG	(47)
	**R**	ACCGCCTGCGCTCGCTTTAC	
E. coli G58	**F**	GCGAGGTAACCTCGAACATG	(48)
	**R**	CGCCGTATCGATAATTCACG	
Rotavirus B	**F**	GGTTTAAATAGCCCAACCGGTGC	(52)
	**R**	TGCAATTTRATGCATGCGTT	
	**Probe**	FAM/AGCATGGATCTGATYGAAACRGT	

Rotavirus C	**F**	ATGTAGCATGATTCACGAATGGG	(51)
	**R**	ACATTTCATCCTCCTGGGGATC	
	**Probe**	FAM/TTGCGTAGGGGCAAATGC	

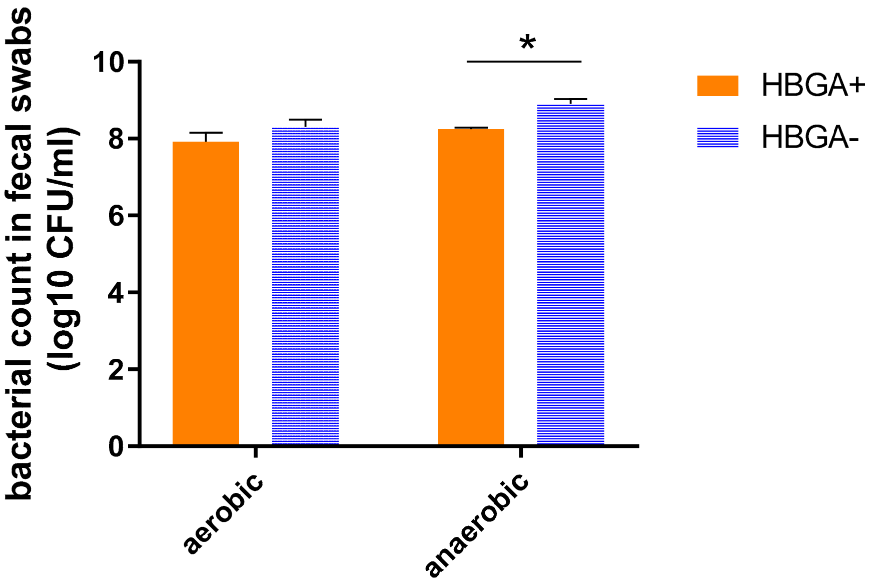

Figure S1. Fecal probiotic bacterial shedding from probiotic colonized piglets. An asterisk indicates significant difference ($p < 0.05$) in fecal probiotics counts among treatment groups.

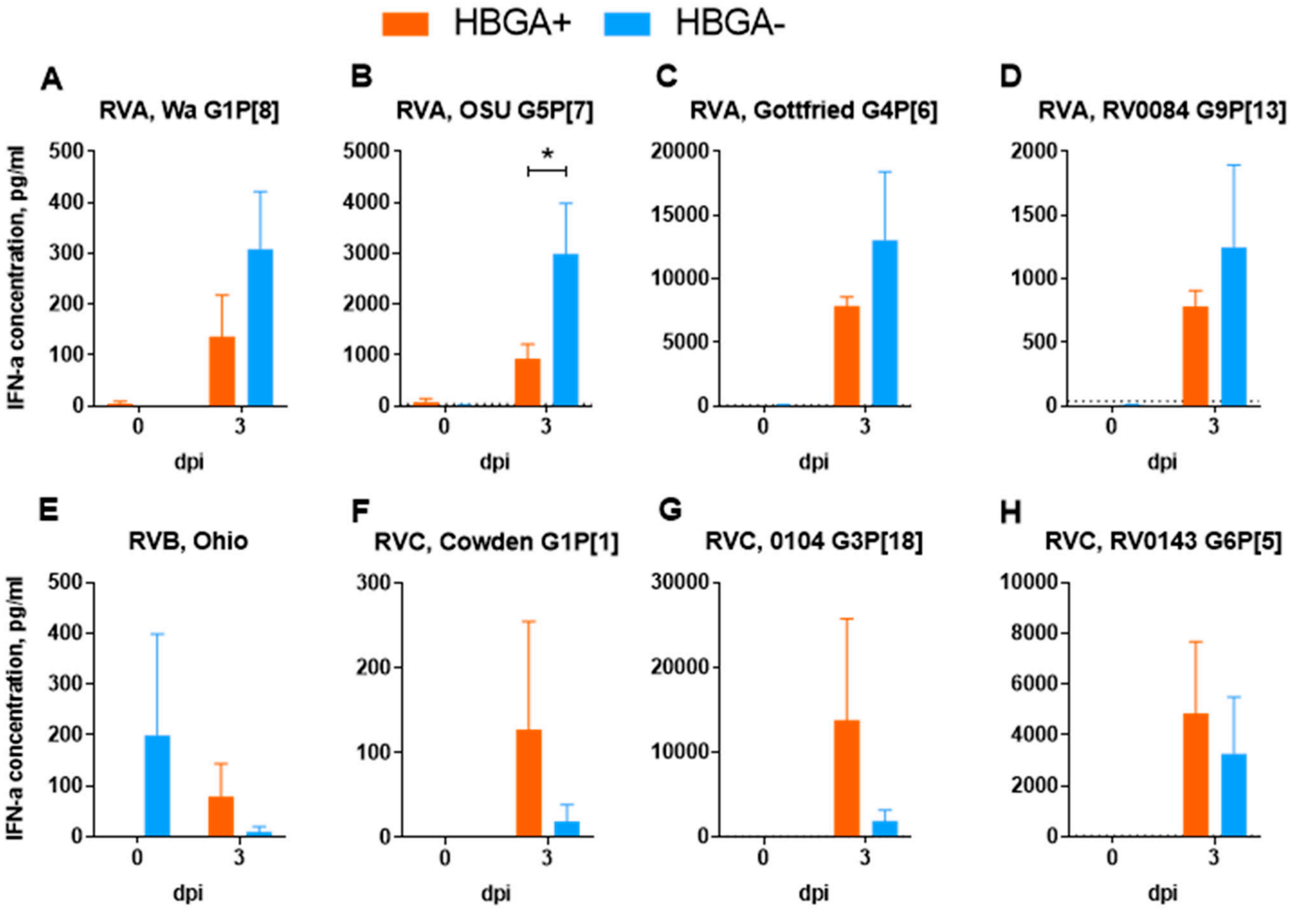

Figure S2. IFN-α concentrations in piglet blood samples collected on dpi 0 and dpi 3 following RVA (A-D); RVB (E) and RVC (F-H) infection. Significant differences (* $p < 0.05$) are indicated as calculated by two-way ANOVA followed by Sidak's multiple comparisons test.

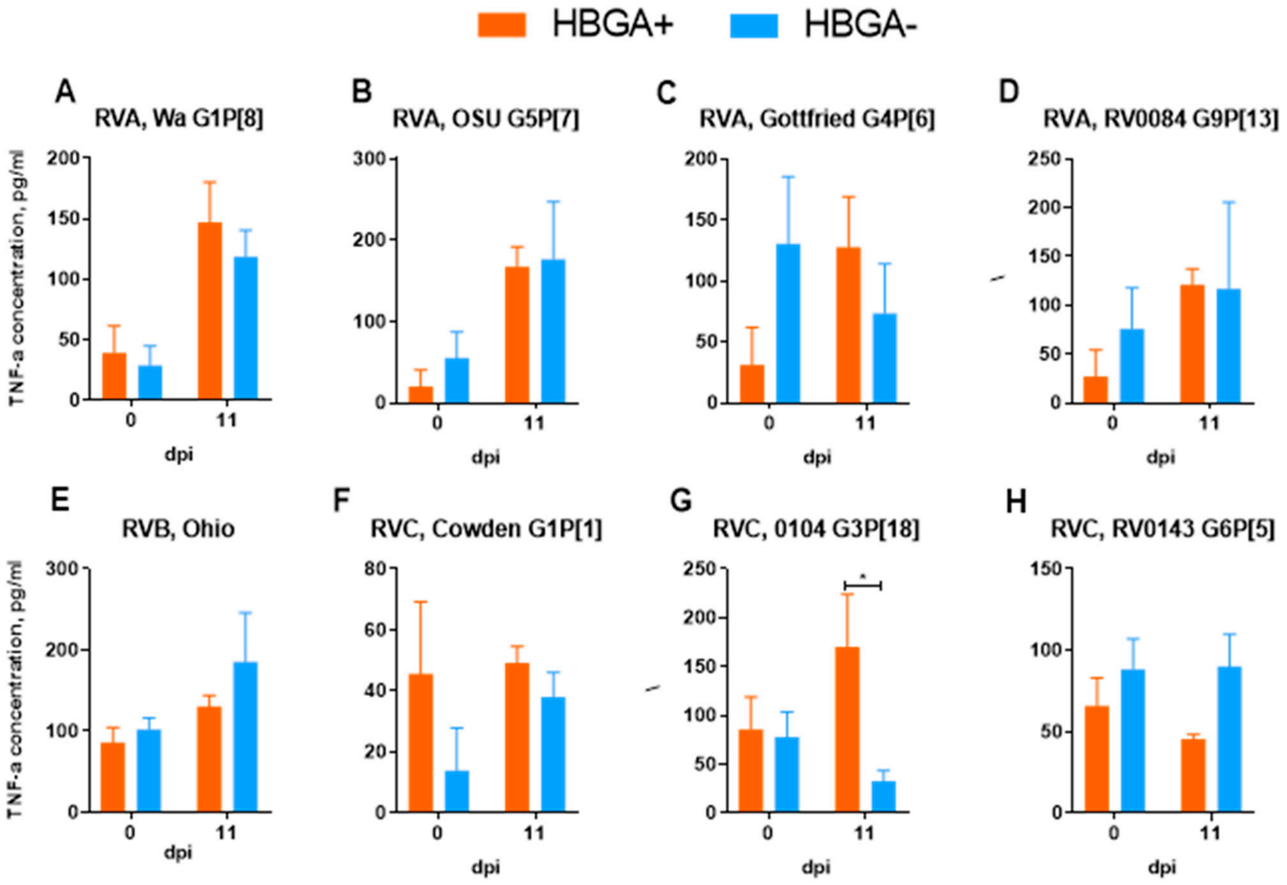

Figure S3. TNF-α concentrations in piglet blood samples collected on dpi0 and 11 following RVA (A-D); RVB (E) and RVC (F-H) infection. Significant differences (* $p < 0.05$) are indicated as calculated by two-way ANOVA followed by Duncan's multiple comparisons test.

Table S2. Diarrhea and virus shedding in gnotobiotic piglets after oral inoculation with virulent RVs.

RV species	HBGA phenotype	N		Diarrhea								Viral shedding							
				Mean days to diarrhea onset[1]		Mean diarrhea duration (days)[2]		Mean cumulative fecal score[3]		AUC[4]		Mean days to shedding onset		Mean shedding duration (days)		Avg peak titer (FFU/ml)		AUC[4]	
		Bacterial cocktail																	
		$HBGA^+$	$HBGA^-$	$HBGA^+$	$HBGA^-$	$HBGA^+$	$HBGA^-$	$HBGA^+$	$HBGA^-$	$HBGA^+$	$HBGA^-$	$HBGA^+$	$HBGA^-$	$HBGA^+$	$HBGA^-$	$HBGA^+$	$HBGA^-$	$HBGA^+$	$HBGA^-$
**RVA**	H^+A^-	3	2	1.7	1.5	3.0	6.5	12	18.5	11	17.7	2	1.5	6	7.5	7.50E+04	1.49E+07	2.41E+06	2.58E+07
	H^+/H^-A^+	12	13	2.3	2	3.7	4.8	12.3	14.5	11.8	14	1.8	1.7	6.3	7.2	4.69E+06	3.30E+06	9.47E+06	6.95E+06
**RVB**	H^+/H^-A^+	4	3	2	1	5.2	7.7	13.5	19.7	13.5	19.7	1	1	7	7	1.05E+03	1.88E+03	2.07E+03	2.44E+03
**RVC**	H^+A^-	5	4	3.8	2.9	4.2	5	14.4	15.5	13.9	14.7	2.4	1.7	8.6	10	2.00E+06	2.00E+05	3.13E+06	3.13E+06
	H^+/H^-A^+	6	11	2.7	2.5	4.2	5.1	13.5	15.5	13	14.9	2	1.4	9.2	10	1.54E+06	7.39E+05	1.71E+06	1.71E+06

[1]Diarrhea onset is defined as the number of days between the virus inoculation and the first manifestation of diarrhea with a score not less than 2. [2]Duration of diarrhea is defined as the number of days that the diarrhea score was 2. Fecal diarrhea was scored as follows: 0, normal; 1, pasty; 2, semiliquid; 3, liquid. [3]Mean cumulative fecal score [(sum of fecal consistency score for 11 days postinoculation)/N], where N is the number of pigs receiving the inoculation. Means in the same row were analyzed by unpaired t-test. [4]Area under the curve (AUC) was calculated using the area under the curve analysis function in the Prism software. A Kruskal-Wallis rank sum test was then performed to compare the total AUC between the groups. Significant differences (**bold**) are indicated as calculated by unpaired t-test.

Table S3. Data on the ability of individual HBGA+ and HBGA- bacterial strains to bind virulent RVs (16).

HBGA expression status	Strain	RV-bacteria binding				
		RVA				RVC
		G5P[7]	G9P[13]	G4P[6]	G1P[8]	G1P[1]
HBGA+	*S. bovis*	no	no	no	no	no
	B. thetaiotaomicron	no	no	no	**yes***	no
	C. clostridiiforme	**yes*****	**yes*****	no	no	no
	B. adolescentis	no	no	**yes***	**yes***	**yes***
	E. coli G58	no	**yes****	**yes*****	**yes****	no
HBGA-	*B. longum*	no	no	no	no	no
	L. brevis	no	no	no	no	no

The table represents the difference in the amount of bacteria-bound RV. * P<0.05, ** P⩽0.01, *** P⩽0.001.
